# The Self and Its Nature: A Psychopathological Perspective on the Risk-Reducing Effects of Environmental Green Space for Psychosis

**DOI:** 10.3389/fpsyg.2020.531840

**Published:** 2020-11-11

**Authors:** Sjoerd J. H. Ebisch

**Affiliations:** Department of Neuroscience, Imaging and Clinical Sciences, Institute of Advanced Biomedical Technologies (ITAB), G. d’Annunzio University of Chieti-Pescara, Chieti, Italy

**Keywords:** intrinsic self, extrinsic self, environment, green space, natural surroundings, psychosis, schizophrenia, urban

## Abstract

Epidemiological studies have shown that environmental green space contributes to the reduction of psychosis incidence in the population. Clarifying the psychological and neuro-functional mechanisms underlying the risk-decreasing effects of green surroundings could help optimize preventive environmental interventions. This perspective article specifically aims to open a new window on the link between environmental green space and psychosis by considering its core psychopathological features. Psychotic disorders, such as schizophrenia, are essentially characterized by self-disturbances. The psychological structure of the self has been described as a multidimensional phenomenon that emerges from the reciprocal interaction with the environment through intrinsic and extrinsic self-processes. The intrinsic self refers to the experience of mental activity and environmental information as inherently related to one’s own person, which involves self-referential processing, self-reflection, memory, interoception, and emotional evaluation. The extrinsic self refers to sensorimotor interactions with the environment and the sense of agency, that is, the experience of being the source of one’s own actions and the multisensory consequences thereof. In psychosis, anomalous self-processing has been related to a functional fragmentation of intrinsic and extrinsic self-processes and related brain networks. Moreover, evidence from cognitive neuroscience suggests that green space could have beneficial effects on self-related processing. Based on the literature, it could be hypothesized that self-processing is involved in mediating the beneficial effects of green space for psychosis. Considering the multidimensionality of the self, it is proposed that urban green space design aimed at improving mental health ideally impacts the complexity of self-facets and thus restores the individual’s self.

## Introduction

Psychotic disorders are severe mental illnesses that involve a “loss of vital contact with reality” (Minkowski, 1927; Parnas and Henriksen, 2014). The most studied and pervasive psychotic disorder is likely schizophrenia, but psychotic episodes can happen in various psychiatric, neurological, and neurodevelopmental conditions and can also be induced by the use of medications or psychoactive drugs. Socioeconomically, psychosis has been associated with premature mortality, morbidity, financial and social burdens, and poor outcomes (Rössler et al., 2005; van Os and Linscott, 2012; Fazel et al., 2014). Psychoses, particularly schizophrenia, place large demands on public healthcare and its budgets (Rössler et al., 2005).

The high heritability of psychosis indicates the existence of genetic risk factors (Smigielski et al., 2020), although genetic components cannot fully explain the development of psychosis (van Os et al., 2010; Pries et al., 2018; Torrey and Yolken, 2019). The risk of developing a psychosis during one’s life is also determined by biological, psychological, and social factors (McGrath et al., 2004; van Os et al., 2010; Fusar-Poli et al., 2017). This knowledge provides important information for intervention programs aimed at detecting high-risk individuals and preventing them from becoming psychotic (Ruhrmann et al., 2010; van Os et al., 2017). However, the implementation of large-scale intervention strategies is still limited. In addition to therapeutic interventions (McGorry et al., 2009; Fusar-Poli et al., 2019), other possibilities for preventive intervention have been implied. Epidemiological studies suggest that environmental factors are involved in the development of psychosis as well (van Os et al., 2010; Dragt et al., 2011; Fusar-Poli et al., 2017). Moreover, longitudinal studies suggest that the modulation of psychosis incidence through environmental factors likely involves causal relationships that cannot be explained by other mediating epidemiological variables (van Os et al., 2010).

Some of these factors may be attractive targets for global risk-reducing environmental interventions (Sussman and Hollander, 2014). Among the various environmental factors that have been related to psychosis risk, urbanicity has been consistently associated with an elevated risk of psychosis as compared to living in rural environments (Lewis et al., 1992; Pedersen and Mortensen, 2001; Sundquist et al., 2004; van Os et al., 2004; Newbury et al., 2016; see for reviews, Krabbendam and van Os, 2005; Kelly et al., 2010). Given that the majority of the world population currently lives in urban areas and continues to increase, this is an issue of concern for mental healthcare (United Nations, Department of Economic and Social Affairs, Population Division, 2014). One notable difference between urban and rural environments that has recently received increasing attention in the scientific literature concerning environmental effects on mental health is the quantity and kind of natural or green space (Verheij et al., 2008), which could be hypothesized as constituting a risk-modulating factor for psychosis. In the present article, I will specifically examine the utilization potential of green space to reduce the incidence of psychosis, although it should be emphasized that the diminished availability of green space is by no means the only characteristic of urban environments and that psychosis incidence in the population is likely modulated by a complexity of interacting nvironmental and genetic factors (Tsuang, 2000; Schmitt et al., 2014).

In particular, this perspective article aims at developing a new hypothesis regarding the psychological and neuronal mechanisms that could explain the risk-decreasing effects of natural surroundings for psychosis. For the purpose of focus, an investigation of how the effects of green space on psychosis incidence can be embedded in the full complexity of genetic and environmental factors and events remains beyond the scope of the present article, although, this will be a mandatory topic for ensuing dialog. A better understanding of the underlying risk-reducing mechanisms of concrete environmental features could help in optimally exploiting these features in urban design. Given the fact that structural and direct investigations in compromising mental disorders like psychosis are still sparse, one approach to exploring these mechanisms would be to link the core psychopathological features of psychosis to green space effects.

## Green Space Exposure is Related to Reduced Psychosis Risk

Natural environments usually refer to surroundings that include green space (essentially typified by the presence of vegetation) but also can include blue space (water bodies), which could be developed with or without human intervention (Völker and Kistemann, 2011; Bratman et al., 2012; Smith et al., 2017). The definition of green space, as used in studies that investigate its psychological effects through experience, covers a broad range of environments, from pristine nature to human cultivated gardens, and more specifically includes urban greens, forests/woodlands, countryside/farmland, and the wilderness (Bratman et al., 2012). Urban green space, in turn, can be characterized by the more formal or informal design of gardens and parks (Twedt et al., 2016). The efficiency of green space exposure in modulating health and well-being has been studied more generally in terms of the presence of green space in people’s living environments and also more specifically in terms of visual exposure (e.g., window views and virtual environments) and physically spending time in or interacting with green space (Bratman et al., 2012).

The beneficial effects of natural surroundings on daily life have been shown for physical and mental health, social well-being, academic and job performance, and happiness (Hartig, 1993; Maas et al., 2009; Bratman et al., 2012; White et al., 2013; Hartig et al., 2014; Cohen-Cline et al., 2015; James et al., 2015; van den Berg et al., 2015; Sarkar et al., 2018). Although the nature of such effects remains to be clarified, natural surroundings, as compared to urban areas, are generally thought to decrease air and noise pollution, improve social cohesion and stress regulation, facilitate the restoration of attention and fatigue, improve the mood, and reduce depressive symptoms (Kaplan and Kaplan, 1989; Ulrich et al., 1991; Berman et al., 2008; Bratman et al., 2012; Markevych et al., 2017; Twohig-Bennett and Jones, 2018). A recent study highlighted the relevance of environmental interventions by showing that individuals frequenting urban environments with few green resources and an increased risk for mental illness are particularly receptive of the presence of urban green space (Tost et al., 2019). Some further evidence suggests that being exposed to real green and blue space induces psychophysiological and behavioral responses that can be differentiated from those induced by the same environments when reproduced by visual media (Huang, 2009; Huang et al., 2019). Similarly, a recent meta-analysis showed that real natural settings benefit mood to a greater degree than simulated settings (Browning et al., 2020).

To date, the relationship between the presence of residential natural spaces and a lower risk for psychosis has been investigated by few studies. Boers et al. (2018) showed that psychotic patients had a significantly lower amounts of available green space (agricultural areas, natural areas, and artificially installed greenery) but not blue space near their residence, while controlling for age, gender, urbanicity, and socioeconomic status. The same study did not find any significant relationship between green space and the length of stay in a psychiatric ward as a measure of illness severity. However, because no other measures of illness severity were included, further studies may need to consider more direct measures as well, including illness impact and symptomatology. A nation-wide, population-based study including more than 900,000 people showed that a decreased presence of green space near one’s residence during childhood (calculated as the normalized difference vegetation index, NDVI, based on remote sensing satellite images) was associated with higher incidence rate ratios for many psychiatric disorders, including schizophrenia and related psychotic disorders (Engemann et al., 2019a). A dose-response relationship over time was also observed in this study, and the lowest levels of green space were associated with up to a 55% higher risk of psychiatric disorders. Additionally, when adjusting for urbanization, parents’ socioeconomic status, family history, parental age, and municipal socioeconomic factors, these effects remained significant for most disorders. Another nation-wide population-based study by the same group focusing on schizophrenia further showed a dose-response relationship between the amount of residential green space present during childhood (NDVI), but not green space heterogeneity, and the risk of developing schizophrenia later in life (Engemann et al., 2018). Also, in this case, the results remained stable after adjustment for urbanization, age, sex, and socioeconomic status. Finally, in a series of follow-up studies, Engemann et al. (2019b) more specifically associated agricultural areas and near-natural green and blue space with lower schizophrenia rates as compared to urban areas, whereas vegetation density (NVDI) was negatively associated with schizophrenia rates in a dose-response manner independently for urban and agricultural areas. Moreover, based on hazard ratios, additive effects on schizophrenia risk were found for childhood green space exposure (NVDI close to residence based on remote sensing satellite images) and genetic liability, while an interaction between these factors could not be detected (Engemann et al., 2020). Taken together, these epidemiological results suggest that the presence of and access to natural surroundings in rural and urban areas may reflect a modulatory environmental factor that contributes to the prevention of psychosis and that green space effects may be disentangled from urbanicity and genetic effects.

## Disrupted Self-Processing is a Core Feature of Psychosis

One of the questions that is derived from these studies is “What are the critical aspects of psychosis that might be influenced by green space?” From a historical, psychopathological perspective, schizophrenia and related psychotic disorders have been described as self-disorders (Raballo et al., 2011). Building on these early insights, more recently, psychosis has been characterized as a complexity of self-disturbances that is generally described by an exaggerated self-consciousness and a disrupted sense of owning one’s personal perceptions and thoughts (Sass and Parnas, 2003; Nelson et al., 2014). Because self-disturbances are often reported as subclinical phenomena that have already occurred prior to the onset of psychosis, remain stable after the attenuation of frank psychotic symptoms, and are highly predictive of conversion to psychosis, self-disturbances are commonly considered to reflect the core features of psychosis (Schultze-Lutter, 2009; Nelson et al., 2012). These properties of self-disturbances make them interesting targets for preventive intervention programs. Indeed, as reviewed above, green space may positively act on the mechanisms underlying psychosis onset during childhood.

To develop a more concrete understanding of the exact nature of self-disturbances in psychosis, it is useful to take a closer look at the psychological structure of the self-concept. The self and its disturbance have been increasingly portrayed from multiple perspectives in psychology and cognitive neuroscience. A major distinction is that between intrinsic and extrinsic self-processes (Gallagher, 2000; Vanhaudenhuyse et al., 2011), which can be traced to the self-aspects of the “Me,” as the conscious person who is known by himself or herself (e.g., “this is me”), and the “I,” who experiences and interacts with the environment (e.g., “I did that”), as described by James (1890). Specifically, intrinsic self-processing refers to the perception of information as belonging to oneself or as personally relevant, which allows self-reflection and experiencing a sense of identity (Damasio, 1999; Northoff and Bermpohl, 2004; van der Meer et al., 2010). This is mainly an internally directed process that integrates stimuli and thoughts with inner information from memory, personal narrative, and interoception and is related to emotional evaluation through the link with transient bodily states (Northoff and Panksepp, 2008; van der Meer et al., 2010; Qin and Northoff, 2011). In contrast, extrinsic self-processing regards the experience of oneself as the source of one’s own actions and their consequences, that is, the perception of oneself as an entity with a sense of agency (Gallagher, 2000; Haggard, 2017). It can be conceived of as an externally directed process based on the experience of one’s intentional control over the environment through action (Jeannerod, 2003; Gallese and Sinigaglia, 2010). Action-related processes that could be involved include multisensory integration, self-monitoring, and sensorimotor predictions (Blanke, 2012). For instance, the integration of predicted somatosensory, motor, visual, and proprioceptive perceptions with the actual multisensory action consequences is crucial for a coherent self-experience during intentional behavior (Frith et al., 2000; van Kemenade et al., 2019).

This literature suggests that the self is composed of distinguishable but complementary processes. Such processes have been associated with largely distinct brain networks (Vanhaudenhuyse et al., 2011). However, in order to permit self-awareness, the networks involved in intrinsic and extrinsic self-processing (e.g., default mode, sensorimotor, attention, and executive networks) likely interact (Molnar-Szakacs and Uddin, 2013), possibly with an additional modulatory role for the salience network in the insula (Menon and Uddin, 2010). Indeed, recent empirical findings from neuroimaging (Di Plinio et al., 2020) suggest that intrinsic and extrinsic self-processes constitute a multidimensional phenomenon that emerges from a bidirectional interaction between an internal self and its external environment, where such interactions could be facilitated by efficient information exchange and integration across intrinsic and extrinsic self-networks in the brain.

Considering psychosis from this perspective, it can be proposed that anomalous intrinsic and extrinsic self-processes, as well as their functional imbalance, could explain self-disturbances. In particular, it has been proposed that the experience of a disrupted sense of self in its reciprocal interaction with the environment in psychosis could be explained by the fragmented functioning of intrinsic and extrinsic self-networks (Ebisch and Aleman, 2016). Furthermore, a biopsychological neural model has been proposed to describe how psychotic symptoms can be explained *via* a disequilibrium of network states and interference between network activities due to impaired auto-excitation and collateral inhibition (Looijestijn et al., 2015). As a consequence, at the phenomenological level, the distinction between internally and externally generated information may blur, leading to impaired self-recognition, depersonalization, and the tendency to experience one’s thoughts, internal speech, or actions as belonging to an external agent or force, rather than oneself (Kircher and Leube, 2003; Nelson et al., 2009; Waters et al., 2012). Because whether and how the beneficial effects of natural surroundings on psychosis can be related to these self-disturbances remain poorly understood, attempts to clarify this issue would require examining the way in which environmental factors associated with natural spaces affect the cognitive and neural mechanisms underlying self-related processing.

## Can Green Space Improve Self-Processing?

To address this particular question in more detail, a literature search was performed for articles empirically investigating the relationship between green space and self-processing. PubMed and Google Scholar were screened for peer-reviewed articles published from January 1990 to June 2020, using the terms “self” AND “nature experience” OR “nature exposure” OR “green space” OR “natural space.” Similar searches were performed for studies considering self-disturbances, using the terms “psychosis” OR “schizophrenia” AND “self” AND “nature experience” OR “nature exposure” OR “green space” OR “natural space.” Articles that resulted from these searches and relevant references cited in those articles were reviewed, and this selection was further completed by searches in the author’s personal files, where articles published in English were included. The literature search results showed that this specific issue received limited attention. After close inspection of the articles’ content, seven empirical studies were found to be relevant to the present perspective article (i.e., Hunter et al., 2010; Lederbogen et al., 2011; Margalit and Ben-Ari, 2014; Bratman et al., 2015; Hayhurst et al., 2015; Fuller et al., 2017; O’Brien and Lomas, 2017), which are discussed below.

A recent systematic review (Mygind et al., 2019) concluded that there is conditional evidence in children and adolescents for a beneficial effect on the part of diversified nature experiences on self-efficacy, a concept closely related to the sense of agency, though this was based on studies with a note regarding the risk of biased results (mountain and lake activities in O’Brien and Lomas, 2017; wilderness therapy intervention in Margalit and Ben-Ari, 2014; outdoor residential experiences in countryside with woodland in Fuller et al., 2017; and developmental sail voyage experiences in Hayhurst et al., 2015, as well as in Hunter et al., 2010). Similarly positive effects were reported for self-esteem, resilience, and academic and cognitive performance, whereas evidence regarding self-concept, problem solving, and mood were inconclusive (see Mygind et al., 2019). In addition, some cognitive neuroscience studies began to provide relevant insights into the cognitive and neural mechanisms underlying the association between green space and self-processing. In a cross-sectional, functional magnetic resonance imaging (fMRI) study involving non-clinical, healthy individuals, Lederbogen et al. (2011) dissociated the impact of urban upbringing and city living on brain activity when participants were exposed to social evaluative stress. Their results showed that current city living was linked to increased amygdala activity, whereas urban upbringing affected the perigenual anterior cingulate cortex. In another fMRI study, Bratman et al. (2015) investigated the link between nature experiences (a 90-min walk in green space consisting of grassland with scattered trees, shrubs, and fauna, as well as views of hills and a bay, vs. an urban walk in a busy city thoroughfare) and rumination in a sample of non-clinical, healthy participants. Relevantly, rumination can be considered a dysfunctional self-process defined as a maladaptive pattern of self-referential thought and self-relational emotions. Behaviorally, the results showed that the nature experiences, as compared to the urban experiences, led to decreased rumination. Neuro-functionally, nature experiences, as compared to urban experiences, led to decreased neural activity (cerebral blood flow) in the subgenual prefrontal cortex and perigenual anterior cingulate cortex during rumination.

Notably, both fMRI studies (Lederbogen et al., 2011; Bratman et al., 2015) linked the ventromedial frontal cortex, specifically, the perigenual anterior cingulate cortex, to urbanicity or green experiences. Of interest, the perigenual anterior cingulate cortex has been identified as a crucial brain structure for many self-related processes that comprise self-continuity, self-consciousness, self-reflection, self-regulation, and self-other similarity (van der Meer et al., 2010; Northoff, 2017; Scalabrini et al., 2019). The perigenual anterior cingulate cortex is located in the default mode network, which is characterized by high levels of activity when individuals are involved in free thought, such as mind-wandering (Northoff et al., 2010). Moreover, the perigenual anterior cingulate cortex is a key node within a network that integrates information from the memory and interoception and is affected by its interactions with the anterior insula, medial temporal regions, and posterior cingulate cortex (Northoff and Panksepp, 2008). Regarding its involvement in psychosis, fMRI experiments applying explicit tasks of self-evaluation reported deficient intrinsic self-processing in the perigenual anterior cingulate cortex and adjacent ventromedial prefrontal cortices in patients with schizophrenia as compared to healthy controls (Modinos et al., 2011; Kühn and Gallinat, 2013; van der Meer et al., 2013; Tan et al., 2015).

## Discussion

**Figure 1:**
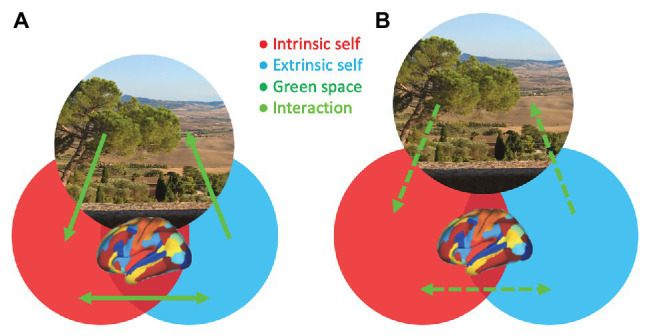
A hypothetical model illustrating the modulation of the integrity of self-related processing, which is impaired in psychosis, through brain network interactions by green space. The experience of natural surroundings (green space area) in one’s living environment (**A**; increased interactions and overlap between green space and self-aspects) or being distantiated from natural surroundings during daily life (**B**; decreased interactions and overlap between green space and self-aspects) is associated with more or less integration of intrinsic (red; internally directed) and extrinsic (blue; externally directed) self-aspects, respectively. The green space photo depicts a view on Val d’Orcia in Tuscany, Italy, an UNESCO World Heritage landscape developed in the 14th and 15th centuries to idealize a well-balanced dialog between humans and nature.

The evidence reviewed above begins to provide new insights into the mechanisms that may explain how green space exposure can reduce psychosis risk. On the one hand, epidemiological studies consistently show that green space exposure decreases the incidence of psychosis, whereas urbanicity is associated with an increased risk for psychosis. On the other hand, cognitive and neuroscience findings preliminarily suggest that exposure to and experiences of green space, as well as blue space, could have beneficial effects on self-related processing. Based on these findings, it is proposed that self-processing and related brain network interactions may constitute a relevant mechanism that mediates between green space exposure and psychosis risk (Figure 1).

Because this hypothesis has received very little or no attention in published empirical investigations using clinical samples and specific reports of positive and null results regarding the link between green space and psychosis-related self-disturbances are lacking, novel studies are encouraged to directly and systematically investigate how the environmental planning of green space could comprehensively facilitate intrinsic and extrinsic self-experiences in non-clinical samples, as well as in psychotic disorders or high-risk populations. The literature reviewed in this article provides concrete perspectives for further investigation. For example, psychological and neuroscientific studies with high levels of control over experimental conditions and modulating factors should reproduce the epidemiological findings. Moreover, definite issues for future studies of the health benefits of green space include the modulating effects of particular types and features of green space and the kind of interactions individuals have with green space on intrinsic/extrinsic self-processing and psychosis risk.

Further limitations of the present review must be mentioned. Because it focuses specifically on the risk-reducing effects of green space for psychosis, it is important to remember that the onset of psychosis depends on a multiplicity of factors and events. The lack of green space exposure is one out of many characteristics of urbanicity that contribute to psychosis risk, in addition to other physical, social, familiar, occupational, and relationship variables (van Os et al., 2010; Brown, 2011; Schmitt et al., 2014). Green space is a piece of an intricated puzzle of interactions between numerous environmental variables, as well as genetic factors, that explains the variance in psychosis onset. It will be crucial for future studies to increase our understanding of how the effects of green space can be embedded in this full complexity of factors, both to explain and prevent psychosis onset (Tsuang, 2000; Howes et al., 2004; Meyer-Lindenberg and Tost, 2012). Finally, in this article, the effect of green space on psychosis prevention is examined in the context of self-disturbances, which are the core features of psychosis and sensitive predictors of its onset, while the relationships with positive symptoms, social impairments, and reduced insight into illness are not considered. It would be relevant to expand this investigating by including these domains too.

In conclusion, self-awareness is a continuous, integrative phenomenon that emerges from a stream of sensory perceptions, actions, bodily states, memories, motivations, thoughts, and imaginations. These processes support a coherent sense of self with a past (autobiographical memories), a present (actual experiences), and a future (prospective thoughts). Green space may be an environment with auspicious qualities that can modulate mental and bodily self-experiences over time and space on various scales, transforming itself continuously as it evolves and changes over time, while at the same time remaining a familiar environment (Kuo, 2015; Northoff and Huang, 2017). It is therefore hypothesized that green space potentially reduces psychosis risk by offering a dynamic environment that within a complex architecture of environmental and genetic factors, supports a stable, multidimensional self-experience over time. Enhancing our knowledge about the exact relationships between green space experiences and multidimensional self-processing would help in environmental planning, especially in urban areas, to optimize the positive impact of green space on mental health and illness prevention.

## Author Contributions

The author confirms being the sole contributor of this work, being responsible for reviewing the literature, developing the concept, and writing the manuscript, and has approved it for publication.

### Conflict of Interest

The author declares that the research was conducted in the absence of any commercial or financial relationships that could be construed as a potential conflict of interest.
